# Microbiota Detection Patterns Correlate With Presence and Severity of Barrett’s Esophagus

**DOI:** 10.3389/fcimb.2021.555072

**Published:** 2021-02-23

**Authors:** Ikenna C. Okereke, Aaron L. Miller, Daniel C. Jupiter, Catherine F. Hamilton, Gabriel L. Reep, Timothy Krill, Clark R. Andersen, Richard B. Pyles

**Affiliations:** ^1^Division of Cardiothoracic Surgery, University of Texas Medical Branch at Galveston, Galveston, TX, United States; ^2^Department of Microbiology and Immunology, University of Texas Medical Branch at Galveston, Galveston, TX , United States; ^3^Department of Preventive Medicine and Population Health, University of Texas Medical Branch at Galveston, Galveston, TX, United States; ^4^Division of Gastroenterology, University of Texas Medical Branch at Galveston, Galveston, TX, United States

**Keywords:** Barrett’s esophagus, microbiome, esophageal cancer, gastroesophageal reflux disease, microbial community

## Abstract

**Background:**

The microbiome has been increasingly associated with different disease processes, but its role in esophagus is largely unknown. Our goal was to determine the associations of the esophageal microbiota with Barrett’s esophagus.

**Methods:**

A total of 74 patients were included in this prospective study, including 34 patients with Barrett’s esophagus and 40 patients without Barrett’s esophagus. Esophageal swabs were obtained from the uvula, and mucosal biopsies were obtained from the proximal esophagus and distal esophagus in each patient. The microbiome of each sample was assessed using a customized Esophageal Microbiome qPCR array (EMB). For each clinical sample, we completed a detection/non-detection analysis for each organism in the EMB. The limit of detection (LOD) for each target was established by analysis of plasmid dilutions.

**Results:**

Average age was 60.2 years. There were significantly different microbial detection patterns in patients with Barrett’s esophagus compared to the control population. There were a greater number of organisms which had different likelihoods of detection in the distal esophagus, compared to the proximal esophagus or uvula. In addition, as the length of the Barrett’s column increased, multiple organisms were less likely to be detected. This decreased likelihood occurred only in the distal esophagus. Beside Barrett’s esophagus, no other demographic factors were associated with differences in detection patterns.

**Conclusions:**

Microbial community structures differ between patients with and without Barrett’s esophagus. Certain organisms are less likely to be detected as the severity of Barrett’s esophagus worsens. These results suggest that particular organisms may have a protective effect against the development of Barrett’s esophagus.

## Introduction

Barrett’s esophagus is a metaplastic change of the distal esophageal mucosa from squamous to simple columnar cells (Theodorou et al., 2012). It is a known risk factor for esophageal cancer (Siersema, 2008; Domper et al., 2015), and patients with Barrett’s esophagus are recommended to undergo periodic surveillance endoscopy examinations (Wani et al., 2016). However, only 1 in 860 patients with Barrett’s esophagus will ultimately develop esophageal cancer (Hvid-Jensen et al., 2011). Therefore, determining which patients with Barrett’s esophagus are at high risk for progression to malignancy would increase the cost effectiveness of surveillance.

The role of the esophageal microbiome in promoting or preventing disease is poorly understood. Several prior studies have shown a relationship between the microbiome and Barrett’s esophagus (Yang et al., 2009; Amir et al., 2014; Snider et al., 2016; Okereke et al., 2019a). However, these articles have been limited and did not examine multiple locations along the esophagus. Our goal was to examine whether the likelihood of detection of particular organisms was affected by the presence and/or severity of Barrett’s esophagus at different locations along the esophagus. Target organisms were identified from the literature and from data created using next generation sequencing of the 16S rRNA gene, followed by the development of a qPCR array (the Esophageal Microbiome Array; EMB) designed to evaluate greater than 85 percent of the detected species or genera in the analyzed samples.

## Materials and Methods

### Study Participants

After institutional review board approval was obtained (IRB # 17-0215), 74 patients were included in the study. All authors had access to the study data and reviewed and approved the final manuscript. All participants were 1) patients undergoing surveillance endoscopy for a known history of Barrett’s esophagus or 2) patients for whom screening endoscopy was recommended or could be considered based on guidelines from the American College of Gastroenterology. Indications for screening included men or women with chronic symptoms (greater than 5 years) of gastroesophageal reflux disease (GERD) and two or more risk factors for Barrett’s esophagus or esophageal adenocarcinoma: Caucasian race, age ≥ 50 years, chronic GERD symptoms, current or prior history of smoking, central obesity as defined as a waist circumference greater than 88 cm, waist to hip ratio greater than 0.8, family history of Barrett’s esophagus or family history of esophageal adenocarcinoma (di Pietro et al., 2015). Patients were enrolled prospectively and consent to participate was obtained voluntarily for each patient. Based on clinical evaluation, individuals were assigned either to the Barrett’s group or the GERD without Barrett’s group.

### Clinical Characteristics

Following endoscopy, physical examination and scripted interviews, the presence of Barrett’s esophagus, age, gender, body mass index (BMI), ethnicity, presence of a hiatal hernia, smoking history and use/dose of proton pump inhibitors were recorded. For patients with Barrett’s esophagus, the presence of dysplasia and the length of the Barrett’s column were also recorded.

### Endoscopy

Prior to its use, the endoscope was sterilized and placed in a sterile container. The endoscope was then removed from the sterile container and placed directly into the esophagus. During the endoscopy, biopsies of the esophagus were taken from 1) normal esophagus from the proximal third of the esophagus (NP) and 2) normal esophagus from the distal esophagus, within one centimeter of the gastroesophageal junction (ND). In patients with Barrett’s esophagus, a mucosal biopsy of normal esophagus was taken within one centimeter of the gastroesophageal junction and adjacent to the Barrett’s esophagus. A swab of the uvula was also obtained using a sterile swab and immediately before the endoscopy was begun.

### DNA Extraction

Once obtained during endoscopy, tissue biopsies were placed into sterile Powerbead tubes pre-loaded with 0.1 mm glass beads (Qiagen, Germantown, MD) plus external lysis buffer in vitro diagnostic (200 µL, Roche Applied Science, Indianapolis, IN). Tissues were homogenized at 30 Hz for 5 min using a Tissuelyser II homogenizer (Qiagen). Swabs from the uvula were placed into sterile PBS, vortexed and then were aliquoted 1:1 into the external lysis buffer (100 µL). Sample lysates were deposited into individual wells of 96 deep-well processing plates. DNA was subsequently extracted in high-throughput fashion using a MagNA Pure 96 instrument running a DNA and viral small volume-in vitro diagnostic extraction kit according to the manufacturer’s protocol (Roche). After extraction, a portion of the DNA was evaluated by Ion Torrent Next Generation Sequencing or using the EMB. The remaining material was archived at −20 °C.

### Ion Torrent Next Generation Sequencing

Sample sequencing was carried out using a fusion-PCR method. Briefly, fusion-primers were designed in accordance with the manufacturer’s guidelines (Ion Amplification Library Preparation – Fusion Method, Life Technologies, Carlsbad, CA) using Ion Xpress Barcodes linked to 16S gene primer pairs targeting hyper-variable regions 1–8 (Klindworth et al., 2013). Each 25 µl PCR was carried out using: 12.5 µl iQ supermix^™^ (Bio-Rad, Hercules, CA), 1 µl of both forward and reverse (5 µM) primers, 9.5 µl nuclease-free water and 1 µl of DNA template. A total of 3 biopools of DNA created by equimolar mixing of the first 5 patient samples were analyzed. Each biopool represented DNA from the uvula swab, the proximal esophageal mucosal tissue or the distal esophageal mucosal tissue. The DNA biopools were then used as templates for creation of subsequent fusion 16s libraries. PCR was completed in a c1000 thermocycler (Bio-Rad) using the following parameters: Cycle 1), 95 C, 3 min, Cycle 2), Step 1: 95 C, 45 s; Step 2: Primer-specific annealing temps., 45 s; Step 3: 72 C, 2 min, repeat 39x; Step 4: 72 C, 7 min. PCR products were purified using Qiagen Qiaquick spin-columns and quantified using a spectrophotometer (Bio-Rad). PCR products were then diluted, mixed in equal proportion and sequenced on an Ion Torrent GeneStudio S5 System using Ion 520 sequencing kits together with 520 size chips following the manufacturer’s instructions (Life Technologies).

### Bioinformatics for Ion Torrent

After generation, sequencing reads were filtered for quality and binned according to Ion Xpress barcode using Ion Torrent Suite software version 5.10.0. Sequencing reads in FASTQ format were further processed using web-based Galaxy software (Blankenburg et al., 2010). First, raw FASTQ files were normalized using the FASTQ groomer tool function. Next, each barcoded read was trimmed to remove the primer sequence and subsequently filtered to the expected size of the 16S gene target. After this level of processing, the sequence reads were concurrently compared to the SILVA 16S database using bowtie 2 software (Langmead and Salzberg, 2012; Yilmaz et al., 2014). This yielded a call to species or genera level as well as the number of times each sequence matched the database (hit-rate). When multiple calls to a genus were made, the number of hits were added accordingly. These numbers were then converted to percentage of total to give an overall ratio of the sequenced sample.

### qPCR Evaluation by Esophageal Microbiome Array (EMB)

**Figure 1:**
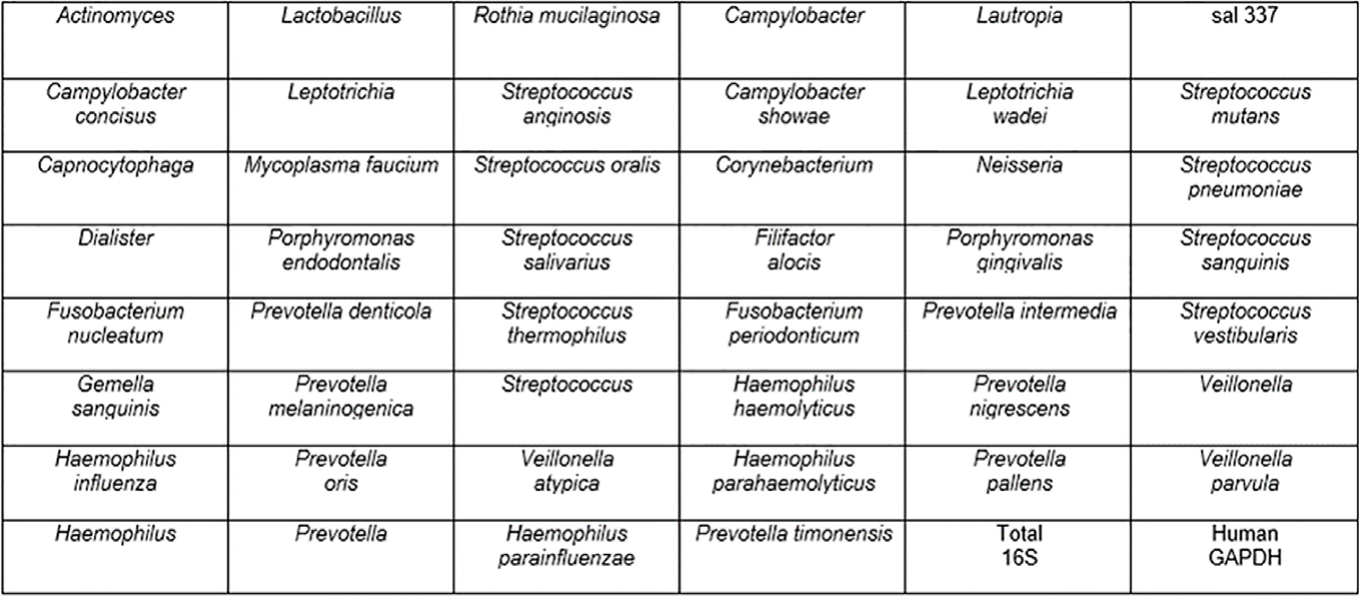
Organisms on EMB array.

To construct the EMB, Ion Torrent data and information from the esophageal disease literature (Paull and Yardley, 1988; Pei et al., 2004; Blackett et al., 2013; Yang et al., 2014; Kaakoush and Morris, 2016; Deshpande et al., 2018; Huang et al., 2018; Okereke et al., 2019b) were compiled to select the most commonly detected organisms from the uvula to the distal esophagus ultimately creating a list of 46 targets that collectively represented greater than 85 percent of the detected microbiota in the Ion Torrent sequencing datasets. Two control qPCR targets were added to address the human DNA (hGAPDH) and total bacterial genomic loads (total 16S) creating a 48 target panel that was constructed in a skirted 96-well plate format (ThermoFisher Scientific Inc.). The 48 target array was constructed in 6 x 8 format allowing for evaluation of 2 samples per 96 well plate (**Figure 1**). Each 25 µl PCR was carried out using: 12.5 µl iQ SYBR green supermix^™^ (Bio-Rad), 1 µl of each forward and reverse (5–10 µM) primer, 9.5 µl nuclease-free water and 1 µl of DNA template. qPCR was completed in a c1000 thermocycler equipped with a CFX^™^ reaction module (Bio-Rad) using the following parameters: Cycle 1), 95 °C, 3 min, Cycle 2), Step 1: 95 °C, 30 s, Step 2: annealing 60 °C, 30 s, extension 72 °C, 30 s repeat 39x, Step 3: 72 °C, 2 min, Step 4: Melt-curve 75–89 °C, 0.2 C temperature increments with 5 s plate read time. Fluorescent signal data were collected at the end of each annealing/extension step. Starting quantity values were extrapolated from standard curves of plasmids harboring the PCR targets previously confirmed by Sanger sequencing. Any organism that was below the limit of detection was categorized as not detected. Mathematical analyses were performed using Excel^™^ (Microsoft Corp., Redmond, WA).

### Plasmid Dilution Verification of Limit of Detection

To determine the limit of detection for each organism in the array, an equal concentration of plasmids representing cloned PCR targets on the EMB array were mixed in a 1:1 ratio. This mixture was 10-fold serially diluted from 10e7 to 10 copies. Five replicate EMB arrays were used for each concentration of the plasmid mix including 10e7, 10e5, 10e3, 10e2, and 10e1. The results were used to establish a dynamic range for quantification as well as the lower limit of detection for each PCR target under the EMB qPCR thermocycling conditions.

### Statistical Analysis

The detection or non-detection of each organism was recorded in every sample in every patient. Two-way Firth-penalized logistic regression was used to relate the detection status to a selected variable (e.g. Barrett’s esophagus vs. GERD without Barrett’s esophagus) separately for each organism at each location. Two-way Firth-penalized logistic regression was used instead of conventional logistic regression due to the extreme values of detection incidence near 0 or 100% in many cases. The graphs for each organism were likewise modeled per 2-way Firth-penalized logistic regression, relating detection status to an association between a group (e.g. Barrett’s esophagus vs. GERD without Barrett’s esophagus) and a location (uvula, proximal esophagus, distal esophagus). The graphs illustrate a model-predicted probability of detection at each location. To determine the association of the length of the Barrett’s column with microbiota, Firth logistic regression was used for detection, restricted to the Barrett’s esophagus group only, controlling for the covariates to determine the association between location and length of the Barrett’s column. Figures were drawn with predictions based on samples with no history of smoking and no esophagitis. Statistical analyses were performed using R statistical software (R Core Team, 2018, version 3.5.1). In all statistical tests, α = 0.05.

## Results

### Demographics

A total of 74 total patients were enrolled in the study, including 34 patients in the Barrett’s group and 40 patients in the GERD without Barrett’s group. Demographic information for each patient is listed in **Table 1**. Demographic data was not available for the first 5 patients in the Barrett’s group. The average age in the entire cohort was 60.2 years. The majority of patients were currently on proton pump inhibitor therapy at the time of endoscopy, including 96% of the Barrett’s group and 85% of the GERD without Barrett’s group. When comparing the Barrett’s group to the GERD without Barrett’s group, there were no significant differences in age, gender ratio, BMI, tobacco use, presence of a hiatal hernia, current use of proton pump inhibitors or dose of proton pump inhibitors.

**Table 1:** Cohort demographics*.

	Barrett’s esophagus	GERD without Barrett’s	p-value
N	34	40	
Male	62% (18/29)	50% (20/40)	0.32
Age, years (mean)	61.7 ± 10.7	59.0 ± 8.9	0.26
BMI (mean)	31.5 ± 8.7	31.1 ± 5.5	0.83
Hiatal hernia	52% (15/29)	35% (14/40)	0.16
Current smoker	24% (7/29)	20% (8/40)	0.61
Current PPI use	97 % (28/29)	88% (35/40)	0.23
Mean PPI dose (mg)	46.2	37.0	0.11

*Demographic data was unavailable in 5 patients in the Barrett’s esophagus group.

### Microbiota Detection Patterns in Patients With and Without Barrett’s Esophagus

**Figure 2:**
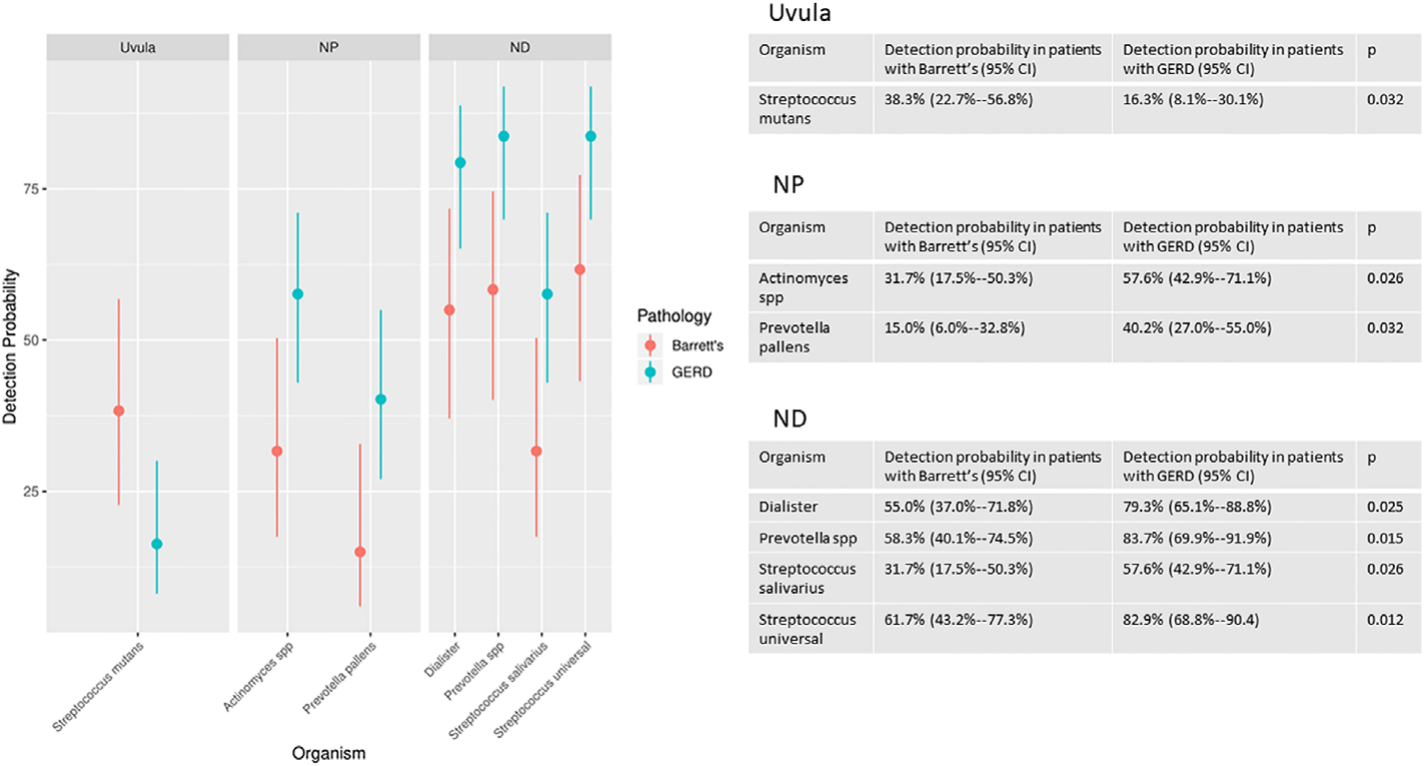
Organisms with significantly different detection rates at the uvula, proximal esophagus (NP) and distal esophagus (ND) in the Barrett’s group compared to the GERD without Barrett’s group.

There were statistically significant differences in the likelihood of detection of multiple organisms in the Barrett’s group compared to the GERD without Barrett’s group. There were significant differences in likelihood of detection in 1 species (*Streptococcus mutans*) at the uvula, 2 genera or species (*Actinomyces, Prevotella pallens*) at the proximal esophagus and 4 genera or species (*Dialister*, *Prevotella unspecified*, *Streptococcus salivarius, Streptococcus unspecified*) at the distal esophagus (**Figure 2**).

### Severity of Barrett’s Esophagus Versus Microbiome Pattern

**Figure 3:**
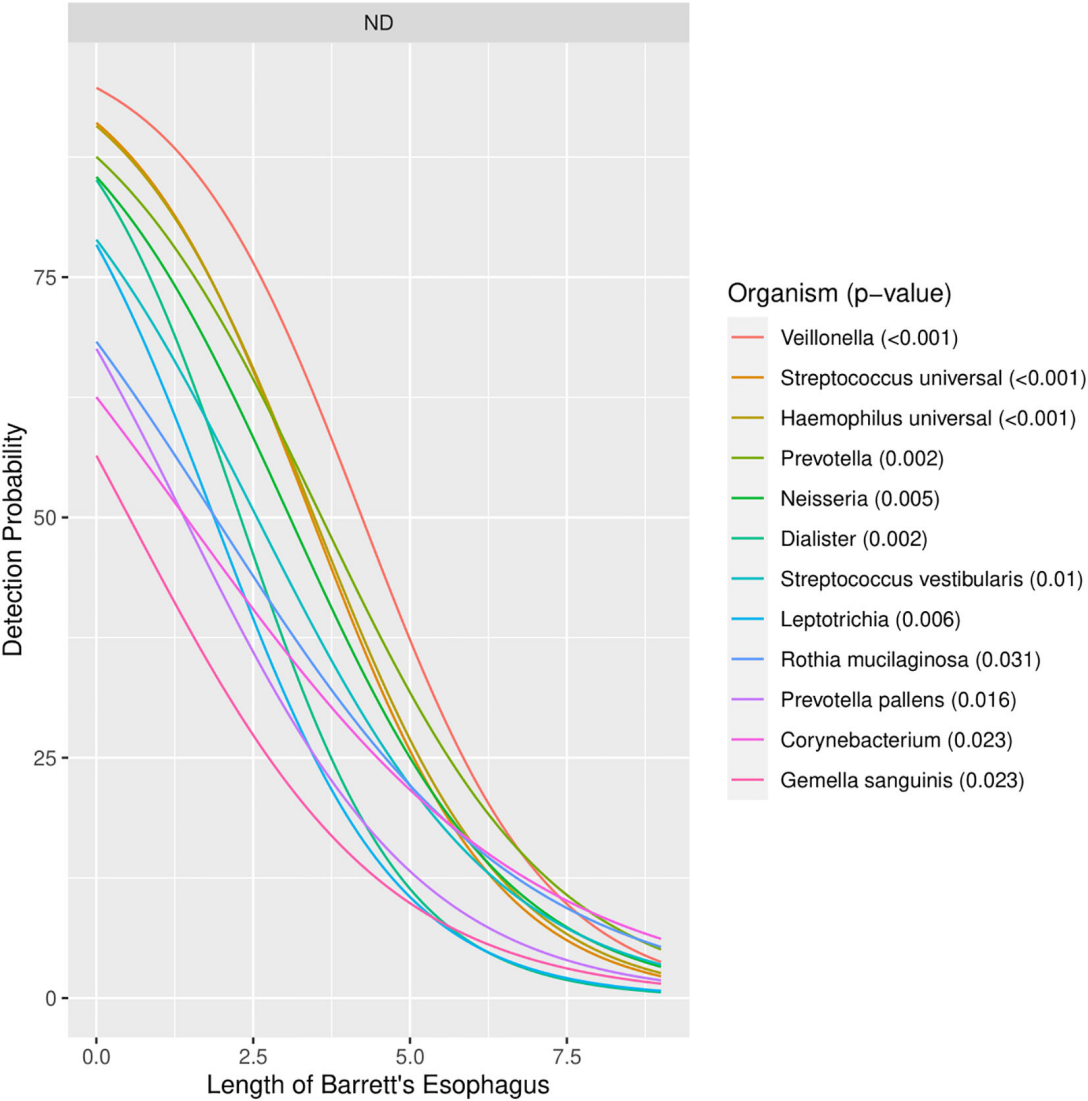
Organisms with detection rates which correlated with length of Barrett’s column.

Among patients with Barrett’s esophagus, the severity of disease was measured by measuring the length of the column of Barrett’s esophagus (Barrett’s column). There was a decreased likelihood of detection of multiple organisms as the length of the Barrett’s column increased. In particular, 10 different genera (*Corynebacterium*, *Dialister, Gemella, Haemophilus, Leptotrichia, Neisseria, Prevotella, Rothia, Streptococcus*, *Veillonella*) on the EMB array had a significantly decreased likelihood of detection as the length of the Barrett’s column increased (**Figure 3**). This relationship between Barrett’s length and detection existed only at the distal esophagus. There was no correlation between Barrett’s length and detection of any organism at the proximal esophagus or the uvula.

**Figure 4:**
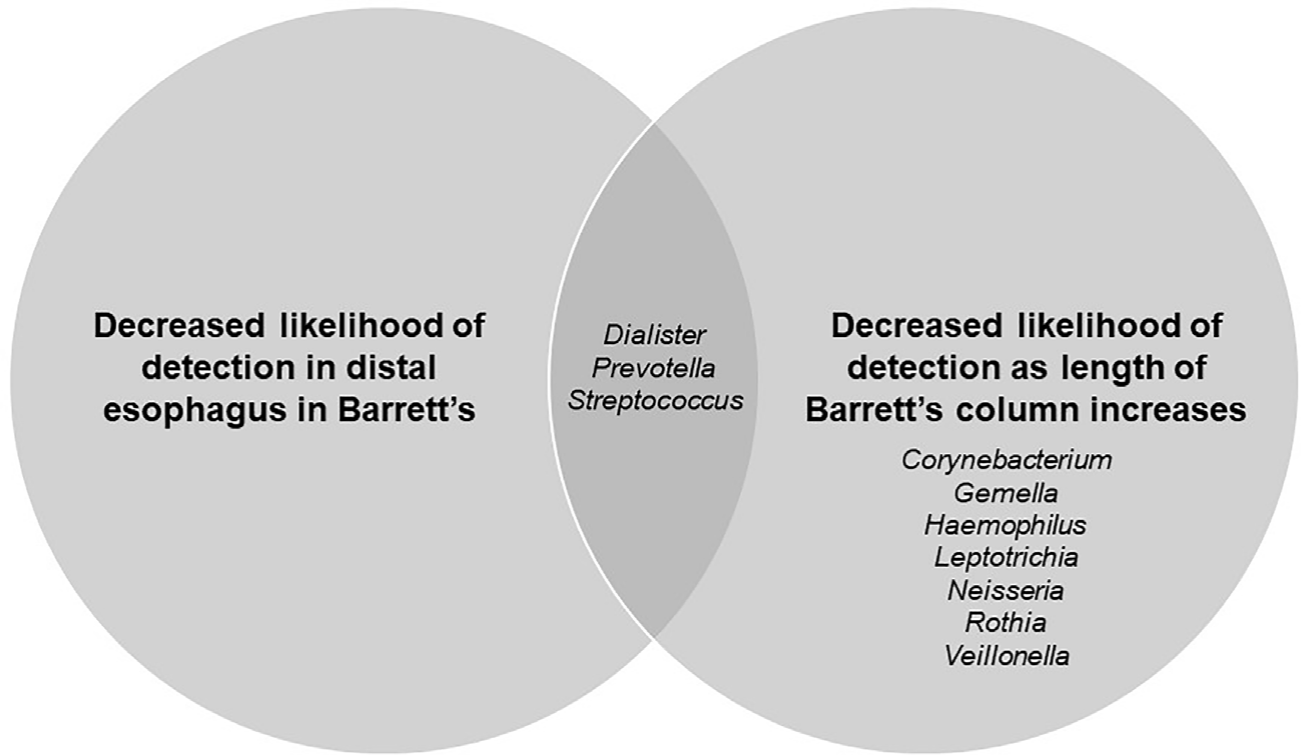
Venn diagram showing genera significantly related to presence/absence of Barrett’s esophagus and length of Barrett’s column.

Finally, there was an overlap in organisms that were significantly associated with the presence or absence of Barrett’s esophagus and the severity of Barrett’s esophagus (**Figure 4**).

### Microbiome Detection Patterns Based on Clinical Factors

No other demographic factors, such as age, gender, body-mass index, geographic location, smoking history or presence of hiatal hernia, were associated with differences in likelihood of detection.

### Limit of Detection (LOD) for Each Target Within the Array

We established the LOD by testing a series of 10-fold dilutions of an equal concentration mix of plasmids containing each PCR target within the range of 10e7 to 10 copies. Five replicate EMB arrays were completed for each concentration establishing a dynamic range for quantification for each PCR target under the EMB qPCR thermocycling conditions. The results indicated that the LOD ranged from <100 to 700 copies of a specific target per tissue or swab sample.

## Discussion

Finding effective screening strategies for solid tumors can be challenging. The main gap in implementing a successful screening plan for esophageal cancer is the relatively low incidence of disease in patients with the known risk factors. Although Barrett’s esophagus is a known risk factor for esophageal cancer, only 0.1% of patients with Barrett’s esophagus develop malignancy. This low incidence for one of the major risk factors highlights the current gap in clinical care that exists for esophageal cancer. This fact highlights the critical need to better stratify the risk of getting esophageal cancer among patients with Barrett’s esophagus as well as the need to identify additional risk factors that have a higher association with incidence of esophageal cancer.

Evaluation of the microbiota on this mucosa is a potential solution to address this gap in clinical care (Zackular et al., 2013; Münch et al., 2019; Okereke et al., 2019c). As such, we undertook this study to determine if there was a microbiota pattern that was associated with Barrett’s esophagus. Our rationale was that a “Barrett’s microbiome” may be able to predict which patients are at risk of developing Barrett’s esophagus, even if they have not yet manifested histologic changes. By the same reasoning, a high-risk microbiome may be able to predict which patients would be most likely to develop esophageal cancer in the future. With this information, screening programs could be tailored more effectively, and a cohort with higher risk could be identified. And the nature of the screening endoscopy could be changed. Currently the endoscopy focuses on the presence or absence of Barrett’s esophagus, the length of the Barrett’s column and the presence or absence of dysplasia. In the future, other information from the screening endoscopy could include the presence or absence of a high-risk community of microbiota (Mosavi-Jarrahi and Mohagheghi, 2006; Richter and Rubenstein, 2018). Finally, if relevant organisms can be associated with an increased risk of disease, then other modalities beside endoscopy could be used. In the future a breath or saliva test may be possible, which would allow for a higher percentage of at-risk patients to be screened at reduced cost and procedure-related risk.

Our study produced two important findings. First, it showed that there was a microbiota community structure that associated with the presence of Barrett’s esophagus. Although this finding has been reported previously, it was interesting and noteworthy in our study that our customized array showed detection differences only for the presence or absence of Barrett’s esophagus, and not for other factors described in other studies such as age, gender or presence of hiatal hernia (Devaraj et al., 2013; Lynch and Pedersen, 2016; Martinez et al., 2017; Kaakoush et al., 2017). The systematic methodology employed to create the customized EMB array helped to make it an appropriate investigative tool for our question and for our ultimate goal of discerning a high-risk community of microbiota. The current data with the 74 patient cohort has identified organisms on the EMB array that are of little consequence to our clinical question that could be replaced as other studies are completed, further refining the array. This is a limitation of the array relative to more standard 16S rRNA gene next generation sequencing. For this focused study we believe that the EMB array offered greater utility than traditional next generation sequencing with sensitivity much higher than that reported for next generation sequencing datasets as well as true quantitation not possible with 16S next generation sequencing. Future studies by our team will investigate organisms not currently on our array that will make use of the archived DNA and original clinical materials produced by this clinical research effort.

Second, our study showed that as the severity of Barrett’s esophagus increased the likelihood of detection of multiple organisms decreased, and that this decreased likelihood was localized to the distal esophagus. To our knowledge, this finding is novel and has not been described in previous literature. Because the length of the Barrett’s column correlates with the likelihood of developing cancer (Hayakawa et al., 2016; Elias and Castell, 2017), these organisms may also be decreased or absent in patients who ultimately develop esophageal cancer. The use of 16S next generation sequencing for this purpose would likely have failed to detect many of these low abundance genera or species. The EMB approach and the data produced in this study raises the possibility that there are multiple organisms that are protective alone or in combination against the development of Barrett’s esophagus and possibly esophageal cancer. It is important to note that the severity of Barrett’s esophagus is based both on the length of the Barrett’s esophagus and on the presence or absence of dysplasia. In patients with Barrett’s esophagus, the prevalence of dysplasia is 11 percent and the prevalence of long segment disease is up to 5 percent (Spechler, 2002; Desai et al., 2019).

Although it is possible that the environmental changes in the distal esophagus that cause Barrett’s esophagus also allow for certain microbes to flourish, there is a distinct possibility that there is a mechanism by which certain organisms act to prevent esophageal disease. We believe that experiments should be performed to investigate any potential means by which certain organisms can interact with the distal esophageal environment to cause or prevent Barrett’s esophagus. If these interactions exist and can be discovered, it is likely that at least some of the same interactions will explain the conversion of normal esophagus to Barrett’s esophagus as well as esophageal cancer. The most obvious and intuitive characteristic of the distal esophagus that may impact the community structure is the acidic nature of the intraluminal milieu at this location. But there are clearly other factors which contribute to the development of disease. For this project, we elected to study a screening group who were evaluated by endoscopy and found to have GERD without Barrett’s as our controls. This meant the added risk of mucosal biopsy for these research purposes was minimal but, more importantly, the controls all had symptoms of GERD. We felt that this control group would also have a rather acidic intraluminal environment in the distal esophagus. Although we did not perform corresponding pH testing in our study, previous literature has not clearly shown that the risk of Barrett’s esophagus increases as the absolute amount of acid exposure increases. Future studies can also evaluate other aspects of the intraluminal environment, such as motility, also affect the risk of development of Barrett’s esophagus. Further studies can also evaluate the role of diet in affecting the esophageal microbiome.

Another major gap in our current understanding of Barrett’s esophagus is how to prevent it from occurring. Although there are modifiable risk factors such as smoking and weight loss in obese patients, the potential to manipulate the esophageal microbiome to be more protective against development of disease would be a major advancement in clinical care. There may be a potential for a probiotic or chemopreventive agent to alter the microbiome to a more favorable one. Shifts in particular organisms have been seen to correlate with the presence of disease in other parts of the gastrointestinal tract, such as the colon (Garrett, 2019). Though previous literature has also identified a microbiome pattern which differs in patients with Barrett’s esophagus compared to patients without Barrett’s esophagus, our study was unique in that we chose to use a detection/non-detection analysis. Other studies have employed a “shotgun” approach in which a much wider array of organisms is used, and diversity indices are reported as differing between the different groups being studied.

Our study design and research approach were directed to creation of a better screening method that might be able to use a detection/non-detection strategy for relevant bacteria. One inherent disadvantage of this strategy, however, is that there is no ability to determine the absolute levels of organisms present, or to determine the importance of relative abundance of certain organisms. We do feel that this information is important, but our immediate goal was to create a focused and appropriate array of organisms to act as a tool for future studies. In the future, our goal is to use the EMB or a second generation array to determine the relevance of the absolute levels of the organisms identified in our study.

Many of the organisms which were shown to be decreased in our Barrett’s group have been seen in other studies as well. Organisms such as *Streptococcus, Prevotella*, and *Veillonella* have been identified as being decreased in other studies (Di Pilato et al., 2016; Snider et al., 2018; Lv et al., 2019), but these other studies largely studied the microbiome at the genera level and evaluated the relative abundance of organisms versus a detection/non-detection technique. Almost certainly species-level information will be important, and our study showed that some species within the same genera did or did not correlate with the severity of disease. As an example, *Streptococcus vestibularis* seemed to correlate with the severity of Barrett’s esophagus while *Streptococcus sanguinis* did not. Although our results from our 74 patient cohort are compelling, future studies into the role of the microbiome in esophageal disease will likely need species-level data to be able to affect clinical behavior and treatment plans.

## Data Availability Statement

The data presented in the study are deposited in the OSFHome repository with the accession number EMB2020_17545.

## Ethics Statement

The studies involving human participants were reviewed and approved by Institutional Review Board of the University of Texas Medical Branch, IRB #17-0215. The patients/participants provided their written informed consent to participate in this study.

## Author Contributions

IO, AM, CH, GR, and RP drafted and edited the manuscript. DJ and CA provided the statistical analysis. IO, AM, DJ, CH, GR, CA, and RP provided the conceptualization of the experiment and the methodology. AM and CH performed the specimen collection and processing. All authors contributed to the article and approved the submitted version.

## Funding

This study was supported by grants from the National Center for Advancing Translational Sciences (NCATS) of the National Institutes of Health, award numbers UL1TR001439 and KL2TR001441.

## Conflict of Interest

The authors declare that the research was conducted in the absence of any commercial or financial relationships that could be construed as a potential conflict of interest.

## Abbreviations

BMI, Body mass index; DNA, Deoxyribonucleic acid; EMB, Esophageal microbiome array; GERD, Gastroesophageal reflux disease; hGAPDH, Glyceraldehyde 3-phosphate dehydrogenase; LOD, Limit of detection; qPCR, Quantitative polymerase chain reaction; rRNA, Ribosomal ribonucleic acid.
